# Interplay between integrins and PI4P5K Sktl is crucial for cell polarization and reepithelialisation during *Drosophila* wound healing

**DOI:** 10.1038/s41598-019-52743-z

**Published:** 2019-11-08

**Authors:** Si-Hyoung Park, Chan-wool Lee, Kwang-Min Choe

**Affiliations:** Department of Systems Biology, Yonsei University, 50 Yonsei-ro, Seodaemun-gu, Seoul, 03722 South Korea

**Keywords:** Collective cell migration, Development

## Abstract

Phosphatidylinositol(4,5)-bisphosphate [PI(4,5)P2] regulates cell adhesion and actin dynamics during cell migration. PI(4,5)P2 binds various components of the cell adhesion machinery, but how these processes affect migration of the epithelial cell sheet is not well understood. Here, we report that PI(4,5)P2 and Sktl, the kinase that converts PI4P to PI(4,5)P2, are both localized to the rear side of cells during wound healing of the *Drosophila* larval epidermis. The Sktl localization requires JNK pathway activation and integrins, but not PVR. The *sktl* knockdown epidermis displays strong defects in would closure, reminiscent of the JNK-depleted epidermis, and shows severe disruption of cell polarity, as determined by myosin II localization. Sktl and βPS integrin colocalize at the rear side of cells forming the trailing edge during wound healing and the two are inter-dependent in that the absence of one severely disrupts the rear localization of the other. These results strongly suggest that the JNK pathway regulates the rear localization of Sktl and integrins and the interplay between Sktl and integrins sets up cell polarity, which is crucial for reepithelialisation during wound healing.

## Introduction

Cell migration occurs through coordinated actions of polarization, protrusion, frontal adhesion, and detachment and retraction of the rear. Components involved in cell adhesion and migration are tightly regulated in a spatiotemporal manner, and the localization of specific molecules to the front or rear side of the cell is one of the most crucial aspects in cell migration because this sets up cell polarity and directionality^1–3^^.^

Among the regulators that affect the behaviour of the cell migration machinery is phosphatidylinositol(4,5)-bisphosphate [PI(4,5)P2]. PI(4,5)P2 is present in the plasma membrane at relatively high concentrations, compared to other phosphoinositides^4^, and is synthesized from PI4P or PI5P by type I or type II phosphatidylinositol phosphate kinases (PIPKs), respectively, or from PI(3,4,5)P3 by PTEN^5,6^. PI(4,5)P2 binds many cell adhesion proteins, including talin and vinculin, and also regulates the actin cytoskeleton by directly binding central actin-binding proteins^7–13^. Most knowledge, however, was obtained in the context of single cell migration, and the possible interplay between PI(4,5)P2, cell adhesion proteins, and their localization in cell-sheet migration has not been investigated well.

Wound healing is mediated by various cellular behaviours of multiple tissue types, but one of the most prominent features is reepithelialisation of cell sheets that cover wound holes^14–16^. Wound healing of the *Drosophila* larval epidermis is an excellent model system to address the molecular mechanisms of cell-sheet migration, because wound healing in this system only involves cell growth and migration, but not proliferation^17^. In the *Drosophila* larva, reepithelialisation of a large wound hole is mediated by at least three different signalling pathways, JNK, PVR, and the Hippo pathways^18–20^. Cell polarization and directionality are set up by a series of small GTPases, Cdc42, Rac1, and Rho1, which collectively mediate the wound signal to JNK and assemble myosin II at the wound margin and at the rear side of individual cells, which is clearly visible in cells that are located in the first three rows from the margin^21–24^. Thus, the rear localization of myosin II is a convenient marker to assess cell polarization.

Integrins are heterodimeric membrane proteins, functioning as a cellular receptor in cell adhesion to the matrix. Integrins have a crucial role in cell migration and consequently in wound healing^25^. In migrating single cells, integrins are often localized to the front side, while being degraded at the rear, which allows the uropod to detach from the matrix. Interestingly, we observed in *Drosophila* larva that αPS2-βPS integrin was localized to the rear side of cells during epidermal wound healing^26^. Without αPS2-βPS and αPS3-βPS integrins, larvae displayed severe defects in wound closure^26,27^. In mammalian cells, the integrin-containing adhesion complex is regulated by PI(4,5)P2 during cell migration^6^. Specifically, talin binds to PI(4,5)P2 via its FERM domain, which targets talin to focal adhesions, and increases affinity for β integrin when analysed in HeLa and NIH3T3 cells^8^. Vinculin binds PI(4,5)P2 via its tail and this appears to promote disassembly of focal adhesions and cell migration of mouse melanoma cells^11^. Whether these types of regulation occur *in vivo*, particularly during cell-sheet migration, has rarely been studied.

Here, we focused on Sktl in *Drosophila* wound healing. The *Drosophila* genome contains two genes that encode the phosphatidylinositol-4-phosphate 5-kinase (PIP5K), which are *sktl* and *dPIP5K*^28^. *sktl* is involved in diverse processes, including vesicle trafficking, setting up apical-basal polarity^29^, and ciliogenesis^30^. *dPIP5K* is essential for re-synthesis of PIP2 in photoreceptors^28^. In the present study, we report that Sktl was localized to the rear side of epidermal cells in a JNK-dependent manner during wound healing. Sktl colocalized with integrins, and the interplay between the two was essential for setting up cell polarity and wound closure.

## Results

### GFP-Sktl and PI(4,5)P2 translocate to the rear side of epidermal cells during wound healing

**Figure 1:**
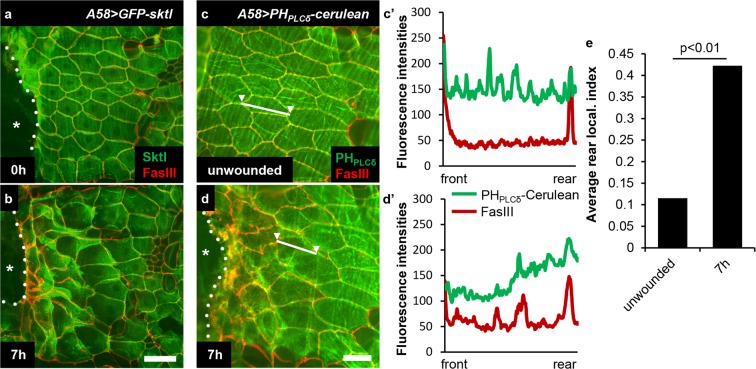
GFP-Sktl and PI(4,5)P_2_ translocated to the rear side of epidermal cells during wound healing. (**a**,**b**) Localization of GFP-Sktl fusion protein, shown in green, after injury. (**a**) 0 h after injury. (**b**) 7 h after injury. (**c**,**d**’) Distribution of PI(4,5)P2, analysed using PH_PLCδ_-Cerulean in unwounded (**c**,**c**’) or wounded epidermis 7 h after injury (**d**,**d**’). (**c**,**d**) Epifluorescence of Cerulean from PH_PLCδ_-Cerulean reporter in green. (**c’**,**d’**) Plotting of the fluorescence intensities along the frontal-rear axis of the cell in regard to the wound, marked with a white line with two arrowheads each in (**c**,**d**). *A58*-*GAL4* is a larval epidermis-specific driver. *A58*-*GAL4 UAS*-*GFP*-*sktl* was abbreviated as *A58* > *GFP*-*sktl*. Cell boundaries were stained with anti-FasIII antibody in red. The asterisk marks the wound hole and the white dotted line indicates the wound margin. Scale bars: 50 μm. (**e**) Rear localization index values for PH_PLCδ_-Cerulean were calculated (see Methods). Mann–Whitney U-test was used for statistical significance.

**Figure 2:**
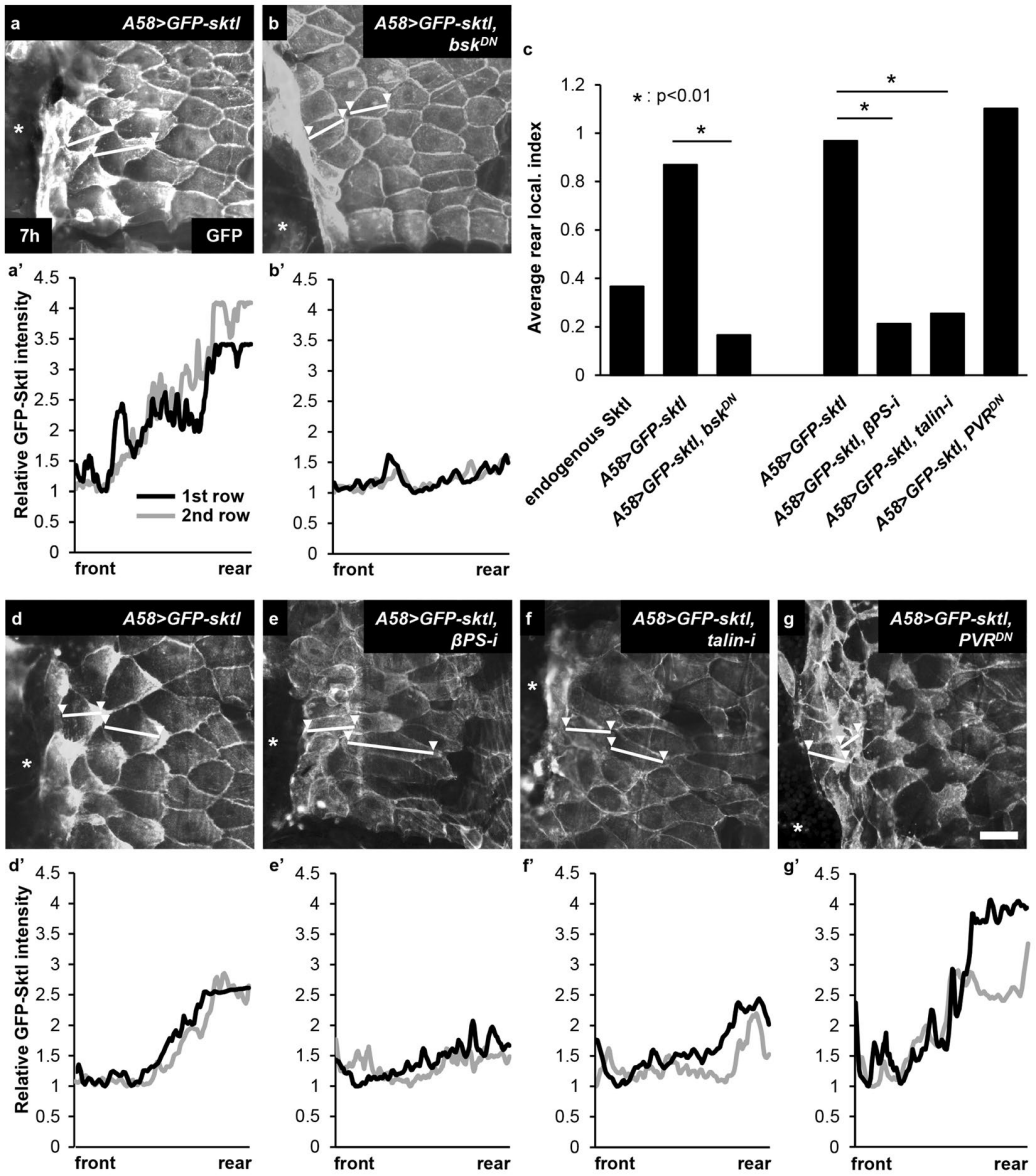
Sktl localization required JNK and integrins, but not PVR. Sktl localization was analysed in the larval epidermis expressing GFP-Sktl, shown in white, 7 h after wounding. (**a**,**d**) Controls. (**b**) *A58* > *bsk*^*DN*^. (**c**) Quantification of the rear localization of GFP-Sktl in the first two rows of cells, presented with the rear localization index value (see Methods for calculation). (**e**) *A58* > *βPS*-*RNAi*. (*A58* > *βPS*-*i*, hereafter). (**f**) *A58* > *talin*-*i*. (**g**) *A58* > *PVR*^*DN*^. The asterisk indicates the wound hole. (**a’**,**b’**,**d**’–**g’**) Plotting of the GFP fluorescence intensities along the frontal-rear axis of the cells in the 1st row (black) and the 2nd row (grey) from the wound margin. The axes were marked in white lines with two arrowheads in (**a**,**b**,**d**–**g**). Scale bar: 50 μm. For statistical analysis, Mann–Whitney U-test was used.

To investigate possible roles of PI(4,5)P2 in *Drosophila* wound healing, we analysed the localization of Sktl protein after epidermal injury generated by pinching the cuticle and abrading approximately 30 epidermal cells. Before or immediately after wounding, the functional fusion protein GFP-Sktl^31^ was evenly distributed in the dorsal epidermal cells of the third instar larva, but translocated to the rear side of the cell during wound healing, which was most evident in the first 2–3 rows of cells from the wound margin. The localization was noticeable 4 h after injury and became distinct by 7 h, the time interval when cells changed their shapes dramatically and migrated to close the wound hole (Fig. 1a,b)^21^. We analysed the endogenous Sktl protein and obtained similar results (see Fig. 2c). We also examined the localization of PI(4,5)P2 using the reporter gene *PH*_*PLCδ*_-*cerulean*^32^. Although the distribution pattern was subtle, the plotting results of the fluorescence intensity of Cerulean across the frontal-rear axis of the cells indicated that the levels of PI(4,5)P2 also increased on the rear side during wound healing (Fig. 1c–e; see Methods for the quantification of rear localization).

### The rear localization of Sktl requires JNK and integrins, but not PVR

To identify the regulators for Sktl localization, we examined Sktl localization in different genetic backgrounds where the signalling pathways critical for wound closure were deficient. In the larval epidermis expressing the gene encoding a dominant negative form of the *Drosophila* JNK (*bsk*^*DN*^), cells remained largely polygonal in shape after injury, as reported previously^18,21^. GFP-Sktl was also evenly distributed across the cell, which was in drastic contrast to wild type (Fig. 2a,b’). We quantified the rear localization of GFP-Sktl based on the GFP fluorescence intensity (Fig. 2c). In the larval epidermis expressing RNAi for integrin *βPS* (*mys*) or *talin* (*rhea*), cells changed their shapes moderately, consistent with the delayed closure of the wound hole^26^, but the rear localization of GFP-Sktl was severely disrupted (Fig. 2c,d–f’). In the larval epidermis expressing the gene encoding a dominant negative form of PVR (*PVR*^*DN*^), GFP-Sktl translocalized to the rear side, which was essentially the same as in wild-type (Fig. 2c,g,g’). Altogether, we conclude that Sktl localization requires JNK and integrins, but not PVR.

### *sktl* is required for wound closure

**Figure 3:**
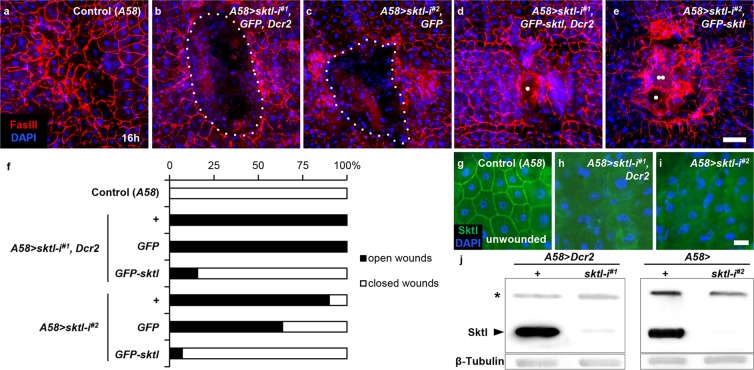
s*ktl* was required for wound closure. Wound closure was analysed 16 h after wounding. (**a**) *A58*-only control. (**b**) *A58* > *sktl*-*i*^*#1*^, *GFP*, *Dcr2*. (**c**) *A58* > *sktl*-*i*^*#2*^, *GFP*. (**d**) *A58* > *sktl*-*i*^*#1*^, *GFP*-*sktl*, *Dcr2*. (**e**) *A58* > *sktl*-*i*^*#2*^, *GFP*-*sktl*. (**f**) Quantification of wound closure. *UAS*-*GFP* was added to balance the *UAS* copy number. Cell nuclei were stained with DAPI in blue and cell boundaries were stained with anti-FasIII antibodies in red. The white dotted line indicates an open-wound hole. The white single dots in (**d**,**e**) indicate scabs induced by breaches on the epidermal-cuticle layer during wounding. The white twin dots in (**e**) indicate large fused cell(s) that form occasionally during wound healing. For each genotype, at least 10 larvae were examined. (**g**–**j**) Confirmation of the knockdown efficiency in unwounded larval epidermis of the indicated genotypes by immunostaining (**g**–**i**) or Western blots (**j**) using anti-Sktl antibody. (**g**) *A58*-*only* control. (**h**) *A58* > *sktl*-*i*^*#1*^, *Dcr2*. (**i**) *A58* > *sktl*-*i*^*#2*^. Sktl protein was visualized using anti-Sktl antibody in green, and the cell nuclei were stained with DAPI in blue. In Western blotting, β-Tubulin was used as a loading control. The asterisk indicates a non-specific band (see Supplemental Fig. S3 for full-length blots). Scale bar: 100 μm (a–e); 25 μm (**g**–**i**).

We examined whether *sktl*-deficient larvae displayed wound closure defects. Because the null mutations of *sktl* are embryonic lethal^31,33^, we knocked down the gene via *UAS*-*RNAi* transgenes using the larval epidermis-specific *A58*-*GAL4*^18^. *sktl* knockdown using two independent *RNAi* lines targeting different regions of *sktl* equally and effectively blocked closure of a wound hole with the size of approximately 30 epidermal cells analysed 16 h after injury, whereas *GAL4*-only control larvae had completely closed the wound hole by that time (Fig. 3a–c,f). We confirmed the result by rescuing the open-wound phenotype by simultaneously overexpressing *UAS*-*GFP*-*sktl* (Fig. 3d–f; *UAS*-*GFP* was added to control groups to balance the *UAS* copy number). We also confirmed that in *sktl*-knockdown larval epidermis, Sktl protein was substantially reduced, as examined by anti-Sktl antibody staining and Western blotting (Fig. 3g–j; Supplemental Fig. 3). Altogether, *sktl* is required for larval epidermal wound closure.

The *sktl* requirement might be ascribed, not to the function of PI(4,5)P2 itself, but to PI(4,5)P2 serving as a precursor for the synthesis of PI(3,4,5)P3, so far, the most versatile and dynamically regulated type of phosphoinositides^6,13^. Alternatively, it might be due to the requirement for diacylglycerol (DAG) and inositol 1,4,5 triphosphate (IP3), which are generated by the hydrolysis of PI(4,5)P2 by phospholipase C (PLC). We examined wound closure in various larvae where the activities of the key enzymes catalysing each of these reactions were depleted and found that the wound holes closed normally when examined at 16 h (Supplemental Fig. S1). Thus, we conclude the *sktl* requirement for wound closure is not due to a lack of PI(3,4,5)P3, DAG, or IP3.

### *sktl* knockdown larvae display disrupted cell polarization during wound healing

We examined further Sktl function in cell polarization during wound healing via the localization of nonmuscle myosin II^21,22^. In wild-type, the myosin II heavy chain Zip and the functional fusion protein GFP-Zip translocate to the rear side of cells 4–8 h after injury, which requires JNK pathway activation^21^.

**Figure 4:**
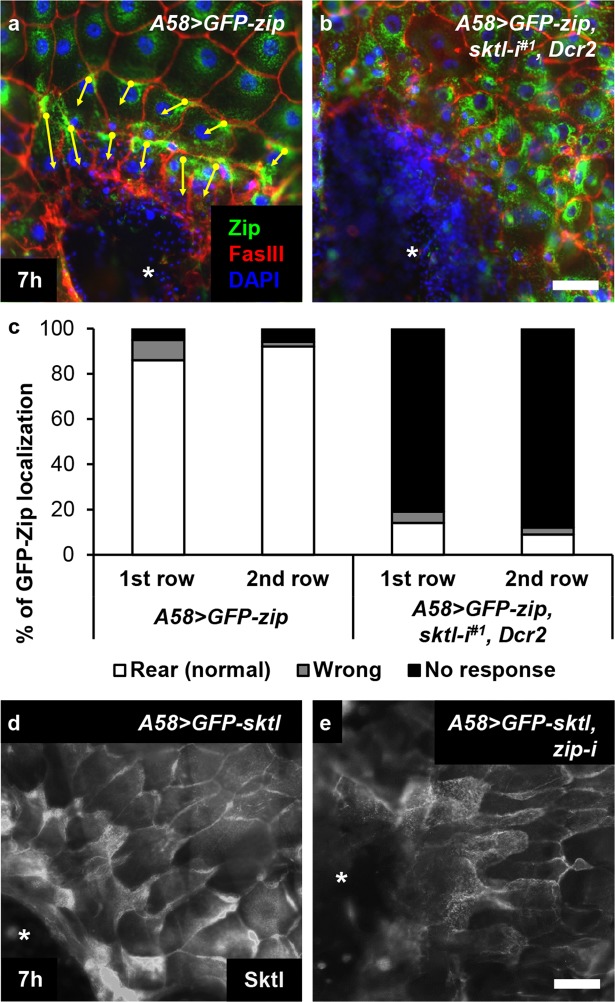
s*ktl* was required for the rear localization of myosin II during wound healing. (**a**,**b**) Localization of nonmuscle myosin II was analysed via GFP-Zip in green 7 h after wounding. (**a**) *A58* > *GFP*-*zip*. (**b**) *A58* > *GFP*-*zip*, *sktl*-*i*^*#1*^, *Dcr2*. Cell nuclei were visualized by DAPI staining in blue and cell boundaries were visualized by anti-FasIII antibody staining in red. The arrows indicate directionality of the cells in the first two rows from the wound margin, based on GFP-Zip localization. (**c**) Quantification of the results in (**a**,**b**). The drastic reduction in the numbers of cells with normal polarization in *sktl*-knockdown larvae is statistically significant (p < 0.01). Mann–Whitney U-test was used. (**d**,**e**) The rear localization of GFP-Sktl protein, shown in white, was analysed in the larvae depleted of the myosin II heavy chain. (**d**) *A58* > *GFP*-*sktl*. (**e**) *A58* > *GFP*-*sktl*, *zip*-*i*. The asterisks indicate wound holes. Scale bars: 50 μm.

In wild-type larvae, 89.1% of the cells located in the first two rows from the wound margin responded correctly to the wound stimulus, as measured by GFP-Zip translocation 7 h after injury (Fig. 4a,c). In *sktl* knockdown larvae, in contrast, merely 11.8% of the cells located within the same distance responded correctly (p < 0.01), and the rest of the cells failed to relocate GFP-Zip; the protein remained mainly in the peri-nuclear region as if the cells did not receive the wound signals (Fig. 4b,c). The cells displaying the wrong directionality were very minor; 5.4% in wild-type and 3.9% in *sktl* knockdown larvae (Fig. 4c).

For epistatic analysis, we performed the reverse experiments, analysing the localization of GFP-Sktl protein in *zip* knockdown larvae. Knockdown of *zip* made larval epidermal cells slightly abnormal and unhealthy, as observed previously^21^, and consequently, the localization pattern of GFP-Sktl was not as distinct as in wild-type. Nonetheless, the rear localization of GFP-Sktl protein was observed in many of the first three rows of cells (Fig. 4d,e). Thus, Sktl, and possibly PI(4,5)P2, are critically involved in setting up cell polarity during wound healing and functions upstream of myosin II.

We also examined whether JNK pathway activation was affected by Sktl depletion. Induction of the JNK pathway reporter *msn*-*lacZ*, analysed 7 h after wounding, was grossly normal in *sktl* knockdown larval epidermis (Supplemental Fig. 2). Therefore, we concluded that Sktl functions downstream of JNK and upstream of myosin II during wound closure.

### *sktl* knockdown larvae are defective in rear localization of βPS integrin during wound healing

**Figure 5:**
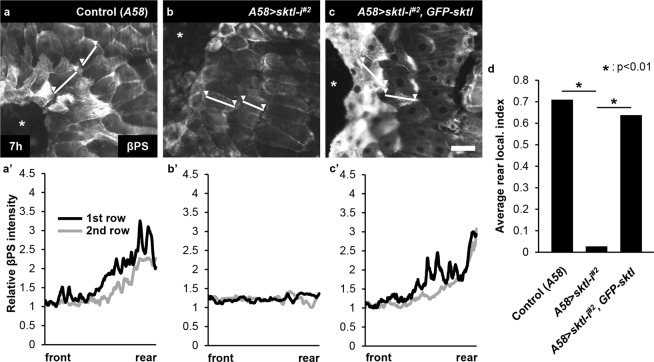
*sktl* was required for the rear localization of βPS integrin during wound healing. βPS localization was examined using anti-βPS antibody staining in white 7 h after wounding. (**a**) *A58*-only control. (**b**) *A58* > *sktl*-*i*^*#2*^. (**c**) *A58* > *sktl*-*i*^*#2*^, *GFP*-*sktl*. The asterisks indicate wound holes. Scale bar: 50 μm. (**a’**-**c’**) Plotting of the fluorescence intensities along the frontal-rear axis of the cells in the 1st row (black) and the 2nd row (grey) from the wound margin. The axes were marked in white lines with two arrowheads in (**a**–**c**). (**d**) Quantification of the rear localization of βPS integrin in the first two rows of cells, presented with the rear localization index value. For statistical analysis, Mann–Whitney U-test was used.

We also assessed cell polarization via immunostaining for βPS integrin, another protein that localizes at the rear side of cells during larval wound healing of the epidermis^26^. In control larvae, βPS integrin localized to the rear side of the cells in the first two rows from the wound margin 7 h after injury, as reported previously (Fig. 5a,a’)^26^. In *sktl* knockdown epidermis, however, βPS integrin appeared evenly distributed in most cells (Fig. 5b,b’). In *GFP*-*sktl*-expressing, knockdown-rescued epidermis, the localization pattern of βPS integrin was also rescued (Fig. 5c,c’). We quantified the results and calculated the rear localization index value (Fig. 5d). Together with the results in Fig. 2e,f we conclude that Sktl and βPS integrin are inter-dependent for their rear localization.

### Sktl and βPS integrin colocalize in the rear side of cells during wound healing

**Figure 6:**
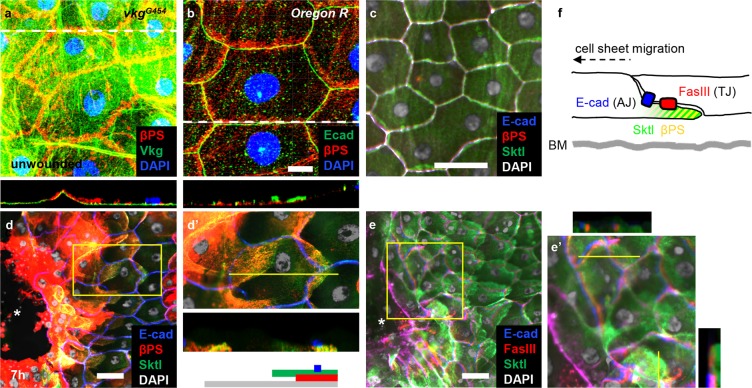
Sktl and βPS proteins colocalized to the rear side of cells during wound healing. Colocalization of Sktl and βPS was examined in unwounded condition (**a**–**c**) or 7 h after wounding (**d**–**e’**). (**a**) *vkg*^*G454*^. (**b**–**e**’) *Oregon R*. (**d**’,**e**’) High-magnification views of the areas marked in yellow boxes in (**d**,**e**). Collagen IV was visualized using the protein trap line *vkg*^*G454*^ in green (**a**). βPS was stained with anti-βPS antibody in red (**a**–**d’**). E-cad was stained with anti-E-cad antibody in green (**b**) or blue (**c**–**e’**). Sktl was stained with anti-Sktl antibody in green (**c**–**e’**). Cell nuclei were stained with DAPI in blue (**a**,**b**) or white (**c**–**e’**). The asterisks indicate wound holes (**d**,**e**). The grey line of the diagram under (**d’**) indicates the length of a cell. The yellow lines in (**d’**,**e’**) indicate the positions of optical sectioning. (**f**) A proposed diagram showing the relative position of each component. AJ: adherens junctions; TJ: tight junctions; BM: basement membranes. Scale bar: 10 μm (b); 50 μm (**c**–**e**).

Because Sktl and βPS integrin both localized to the rear side of cells during wound closure, we directly analysed their colocalization in unwounded samples and wounded samples 7 h after injury, the time point when their rear localization became most prominent (Fig. 1; data not shown)^26^. First, βPS localized on the basal side of the epidermal cells facing the basement membrane, visualized using Vkg-GFP (Fig. 6a), and E-cad was on the lateral side, presumably marking the adherens junction (Fig. 6b,c)^34^. In wounded epidermis, βPS integrin and Sktl colocalized to the rear part of cells that appeared the trailing edge (Fig. 6d,d’). E-cad protein was maintained laterally (Fig. 6d’,e’), whereas the rear part of the cell overlapped with the front part of the cells in the next row, as if the cells lying behind crawled on the cells ahead (Fig. 6d,e’). Consistently, anti-E-cad staining and anti-βPS staining did not overlap in this region; anti-Sktl and anti-βPS staining visualized the presumptive trailing edge, whereas anti-E-cad staining tended to display pentagonal or hexagonal shapes of the cell (Fig. 6d,e’). Localization of FasIII, a septate junction protein that is more basally located, compared to E-cad (Fig. 6e’) similarly marked polygonal shapes of cells (Fig. 6e,e’). Thus, in the rear part of the cell, the region demarcated by E-cad and βPS integrin seems to form the trailing edge, and this was the region with concentrated βPS integrin and Sktl (Fig. 6f).

## Discussion

Here, we report that *sktl*, encoding a *Drosophila* PI4P5K, is required for wound closure of the larval epidermis. Sktl and βPS integrin help each other colocalize to the rear side of cells during wound healing, and this interaction is crucial for setting up cell polarity, which leads to the rear localization of myosin II and reepithelialisation.

We showed, here and previously, that JNK was upstream of these events, as depletion of JNK activation led to disruption of all the events^21,26^. On the other hand, JNK pathway activation was normal in *sktl*, *βPS*, *talin*, or *zip* knockdown larvae. *sktl* or *βPS* knockdown larvae showed disruption in myosin II localization, but *zip* knockdown did not affect Sktl localization. Thus, wounding in the larval epidermis generates as-yet unknown signals that activate the Rho-family small GTPases^22^, which may be activated differentially in the front and the rear sides of the cell, similar to that in single cell migration^2,3^; this leads to the activation of JNK^22^, which is translated to the rear localization of Sktl and βPS integrin^26^.

An interesting question is then the mechanism of how JNK signalling relays the information of cell polarity. In a wounded field, JNK signalling is activated in a graded manner, waning towards the distal area, as analysed by the induction of *msn*-*lacZ* or *puc*-*lacZ*^18^. It is thus plausible that JNK itself provides the frontal-rear information in individual cells, based on the minute difference in JNK activation levels across the frontal-rear axis of individual cells, and possibly, via mutual inhibition between the front and the rear^2^. Alternatively, it may be that a missing factor provides such information and JNK is merely an essential bystander.

We observed that both Sktl protein and PI(4,5)P2 localized to the rear side, but did not provide data to argue strongly that it is PI(4,5)P2 that executes setting up the cell polarity and possibly integrin regulation. One way to address this issue would be to carefully perturb PI(4,5)P2 levels or to swap the wild-type Sktl with a kinase-dead version. Sktl is involved in various processes including cell viability, cytoskeletal organization of actin and microtubule, and polar transport of mRNA, proteins, and vesicles^31,33,35–37^, and the PI(4,5)P2 requirement was nicely shown in some of these cases^35,36^. PI(4,5)P2 interacts with talin, vinculin, moesin, myosin-X, and factors involved in cytoskeletal reorganisation^6^. We, therefore, favour the hypothesis that Sktl and the integrin-containing adhesion complex interact with each other to localize to the rear, and the resulting increase of the local concentration of PI(4,5)P2 affects the behaviour of the adhesion complex and promotes epithelial wound closure.

Lastly, it is worth mentioning some unresolved questions in our study. The first is the question of how PI(4,5)P2 affects the integrin adhesion complex and ultimately reepithelialisation. Interaction between talin and PI4P5K or PI(4,5)P2^38,39^ enhances talin binding either to β integrin in mammalian mesenchymal cells^6,8^ or to myosin II in *Dictyostelium*^40^. These results are, however, inconsistent with our observation that focal adhesion-like structures containing talin were not particularly enriched in the posterior of the cells during reepithelialisation (data not shown). Thus, the two systems must work differently, and a more careful investigation of the function of PI(4,5)P2 may be necessary. Second, our results showing that the presumptive trailing edge of the cells underlies the frontal part of the cells behind are intriguingly different from what has been observed in the migration of MDCK cell sheets, in which the forward protrusion of so-called cryptic lamellipodia of submarginal cells contributes to cell-sheet migration^41^. Future studies should explore the function of the rear localization of PI(4,5)P2, PI4P5K, and integrins, as regulation of PI4P5K activity might be intimately related to various diseases including cancer metastasis^42–44^.

## Methods

### Fly strains

The following stocks were obtained from the Bloomington Stock Center: *msn*-*lacZ* (*msn*^06946^; 11707), *UAS*-*sktl*-*RNAi* (27715), *UAS*-*bsk*^*DN*^ (6409), *UAS*-*PVR*^*DN*^ (58430), *UAS*-*GFP*.*nls* (4776), *UAS*-*Dcr2* (25706), *UAS*-*Pi3K92E*^*A2860C*^ (8289), and *UAS*-*Pten*-*RNAi* (8549, 8550). The following stocks were obtained from the Vienna *Drosophila* RNAi Center: *UAS*-*sktl*-*RNAi* (101624), *UAS*-*zip*-*RNAi* (7819), *UAS*-*norpA*-*i* (105676), *UAS*-*sl*-*i* (108593), and *UAS*-*PLC21C*-*i* (26558). The following stocks were obtained from the National Institute of Genetics: *UAS*-*βPS*-*RNAi* (1560R-1) and *UAS*-*talin*-*RNAi* (6831R-1). The following stocks were obtained from private lab collections: *A58*-*GAL4*^18^, *UAS*-*GFP*-*sktl*^31^, *UAS*-*PH*_*PLCδ*_-*cerulean*^32^, *UAS*-*GFP*-*zip*^45^, *norpA*^*P24*^^46^, and *vkg*^*G454*^.

### Wounding and immunohistochemistry

Third instar larvae were wounded on the dorsal side of segment A2 or A3 by gently pinching the epidermis/cuticle with a pair of forceps (Fine Science Tools, Cat. No. 11295-00). Larvae were incubated on cornmeal-agar media for wound healing. The larval epidermis was dissected in phosphate-buffered saline (PBS) and fixed in 4% paraformaldehyde for 15 min.

Fixed samples were washed three times in [PBS plus 0.5% Triton X-100 (PBST) supplemented with 5% normal goat serum (Gibco, Cat. No. 1913391)] (PBST-NGS) and pre-incubated with PBST-NGS for 1 h. Samples were then incubated with primary antibodies diluted in PBST-NGS overnight at 4 °C. The following primary antibodies were used: mouse anti-Fasciclin III [1:50 dilution; Developmental Studies Hybridoma Bank (DSHB), Cat. No. 7G10], mouse anti-βPS (1:50 dilution; DSHB, Cat. No. CF6G11), mouse anti-β-galactosidase (1:100 dilution; DSHB, Cat. No. JIE7), rat anti-E-cadherin (1:50 dilution; DSHB, Cat No. DCAD2), and rabbit anti-Sktl (1:100 dilution)^29^. Samples were washed in PBST-NGS three times for 10 min and incubated with secondary antibodies diluted in PBST overnight at 4 °C. The following secondary antibodies were used: Cy3-conjugated goat anti-mouse IgG (1:100; Jackson ImmunoResearch, Cat. No. 75512), Alexa 488-conjugated goat anti-mouse IgG (1:200; Molecular Probes, Cat. No. A11001), Alexa 488-conjugated goat anti-rabbit IgG (1:200; Molecular Probes, Cat. No. A11008), Alexa 488-conjugated goat anti-rat IgG (1:200; Molecular Probes, Cat. No. A11006), Alexa 546-conjugated goat anti-rabbit IgG (1:200; Molecular Probes, Cat. No. A11010), and Cy5-conjugated goat anti-mouse IgG (1:100; Jackson ImmunoResearch, Cat. No. 72032). After washing in PBST-NGS five times for 10 min each, samples were mounted on a slide glass using Vectashield (Vector Laboratories, Cat. No. H-1000) and subjected to fluorescence microscopy (ZEISS Axio Imager 2) or confocal microscopy (ZEISS LSM 880). Cell nuclei were stained with 4’,6-diamidino-2-phenylindole (DAPI; Molecular Probes, Cat. No. D1306) at 1:500 dilution.

### Quantification of the rear localization of PH_PLCδ_-Cerulean, Sktl-GFP, Sktl, and βPS integrin

The fluorescence intensity of Cerulean, GFP, or other fluorophores from immunohistochemical staining was measured along the frontal-rear axis of a marginal cell in the wounded area using ImageJ 2 A. Upon determining the frontal-rear axis, the longest line that lied within ± 45° from the hypothetical perpendicular line towards the wound centre was chosen. The rear localization index was calculated as follows: (the intensity value of the rear half of the cell − that of the frontal half) divided by the intensity value of the frontal half. At least > 80% of the total cells located in the first and second rows from the wound margin were measured, which removed non-analysable cells due to damage or other abnormal conditions. At least six larvae were examined per genotype. For the case of PH_PLCδ_-Cerulean, at least > 70% of the total cells located in the first and second rows from the wound margin were examined in four larvae.

### Quantification of GFP-Zip localization

The localization of GFP-Zip was examined in the first two rows of cells from the wound margin. A line with an arrowhead was drawn in each of these cells by placing its arrowhead on the nucleus and the endpoint on the middle of GFP-Zip aggregation. If the arrow had a direction that was within ± 45° from the hypothetical wounded centre, the cell was sorted as ‘normal’. If the arrow was directing outside of the range, the cell was sorted as ‘wrong’. If GFP-Zip remained as if the cell did not receive wound signals (similar to the cells in unwounded samples), the cell was sorted as ‘no response’. At least 70% of the total cells located in the first and second rows from the wound margin were in analysable conditions, and eight larvae were examined.

### Western blot analysis

Larval epidermal samples were boiled in sodium dodecyl sulfate (SDS) sample buffer [250 mM Tris-HCl (pH 6.8), 0.5 M dithiothreitol, 10% SDS, 0.25% bromophenol blue, and 50% glycerol] for 5 min, subjected to 10% SDS-polyacrylamide gel electrophoresis, and were transferred to nitrocellulose membranes. The membranes were blocked with 5% skim milk in TBST [10 mM Tris (pH 8.0), 150 mM NaCl, and 0.05% Tween 20] for 1 h, and probed with primary antibodies at 4 °C overnight. The following primary antibodies were used: rabbit anti-Sktl (1:500 dilution)^29^, and goat anti-β-Tubulin (1:1000 dilution; Santa Cruz Biotechnology, Inc.). The membranes were washed three times in TBST and incubated with HRP-conjugated secondary antibodies [1:1000 dilution; anti-rabbit (Cat. No. sc-2004), and anti-goat (Cat. No. sc-2056)] in TBST with 1% skim milk for 1 h. After washing in TBST three times, the membranes were visualized using the WEST-ZOL Plus® Western blot detection system (iNtRon, Cat. No. 16024).

## Supplementary information

Supplementary Information
