# Supplementary material for: Interplay between integrins and PI4P5K Sktl is crucial for cell polarization and reepithelialisation during Drosophila wound healing
Source: Sci Rep. 2019 Nov 8;9:16331. doi: 10.1038/s41598-019-52743-z (PMC6842001; doi:10.1038/s41598-019-52743-z)
Supplement: Supplementary file 1 — Supplementary Information [file 41598_2019_52743_MOESM1_ESM.docx]

**SUPPLEMENTARY INFORMATION**

**Interplay between integrins and PI4P5K Sktl is crucial for cell polarization and reepithelialisation during *Drosophila* wound healing.**

Si-Hyoung Park^1^, Chan-wool Lee^1^, and Kwang-Min Choe^*^

^*^ Corresponding author (kmchoe@yonsei.ac.kr)

^1^ These two authors contributed equally.

Department of Systems Biology, Yonsei University, 50 Yonsei-ro, Seodaemun-gu, Seoul 03722, South Korea

**CONTENTS**

1. Supplemental Figures S1-S3 Page 2-4

2. Supplemental Methods Page 5

3. Supplemental References Page 5

**SUPPLEMENTAL FIGURES**

**
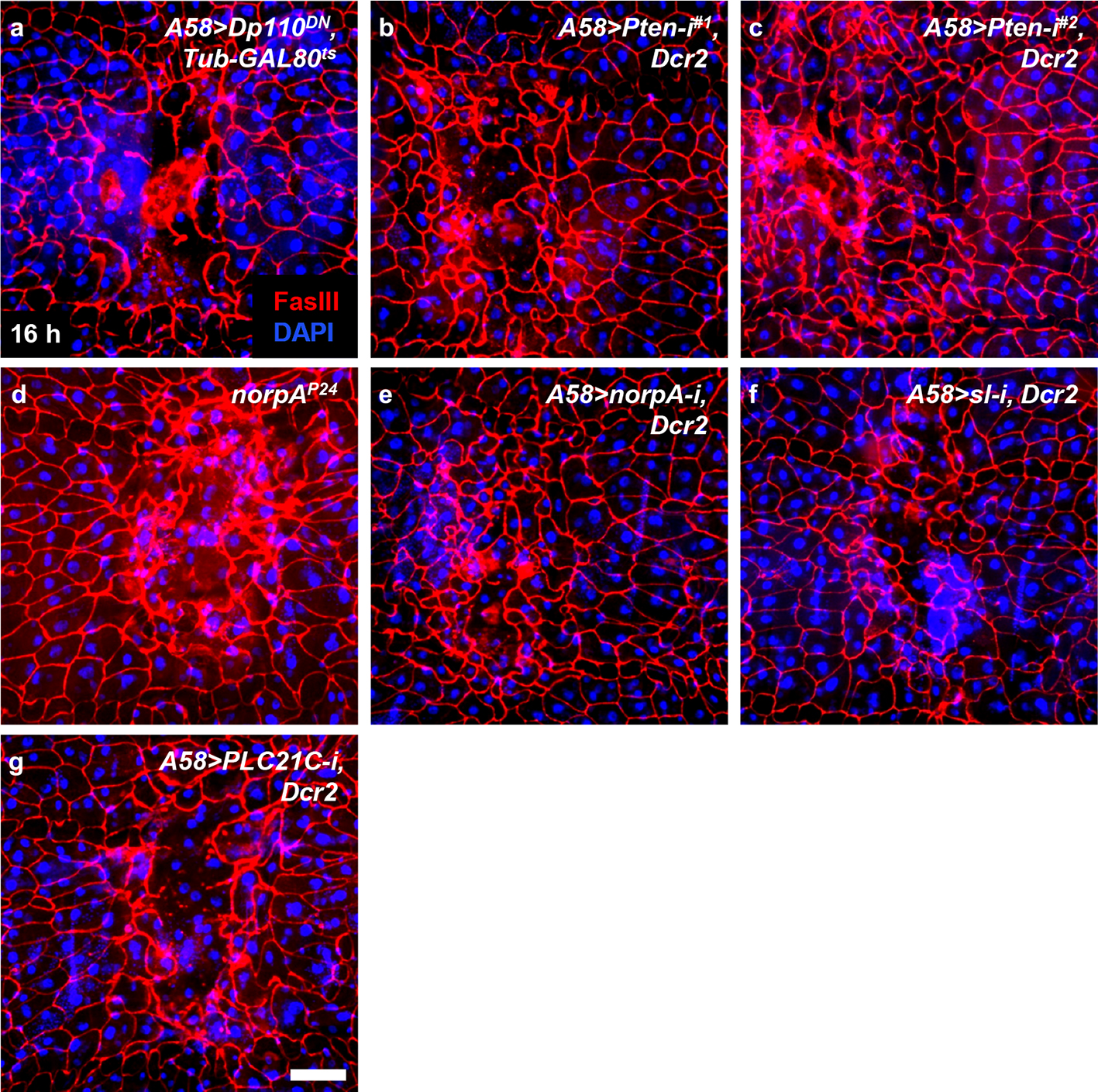
**

**Supplemental Figure S1. Wound closure analysed in the larvae of various genotypes 16 h after injury.** (a) *A58>Dp110^DN^, Tub-GAL80^ts^* (this is *Pi3K92E^A2860C^*, a dominant negative allele). (b) *A58>Pten-i^8549^, Dcr2*. (c) *A58>Pten-i^8550^, Dcr2*. (d) *norpA^P24^* (this is the protein-null allele for PLCβ)^1^. (e) *A58>norpA-i^105676^, Dcr2*. (f) *A58>sl-i^108593^, Dcr2* (*sl* encodes PLCγ). (g) *A58>PLC21C-i^26558^*, *Dcr2*. Cell nuclei were stained with DAPI in blue and cell boundaries were stained with anti-FasIII antibodies in red. For (a), heat shock was given at 30°C for 16 h immediately after injury. For each genotype, at least six larvae were examined. Scale bar: 100 μm.

**
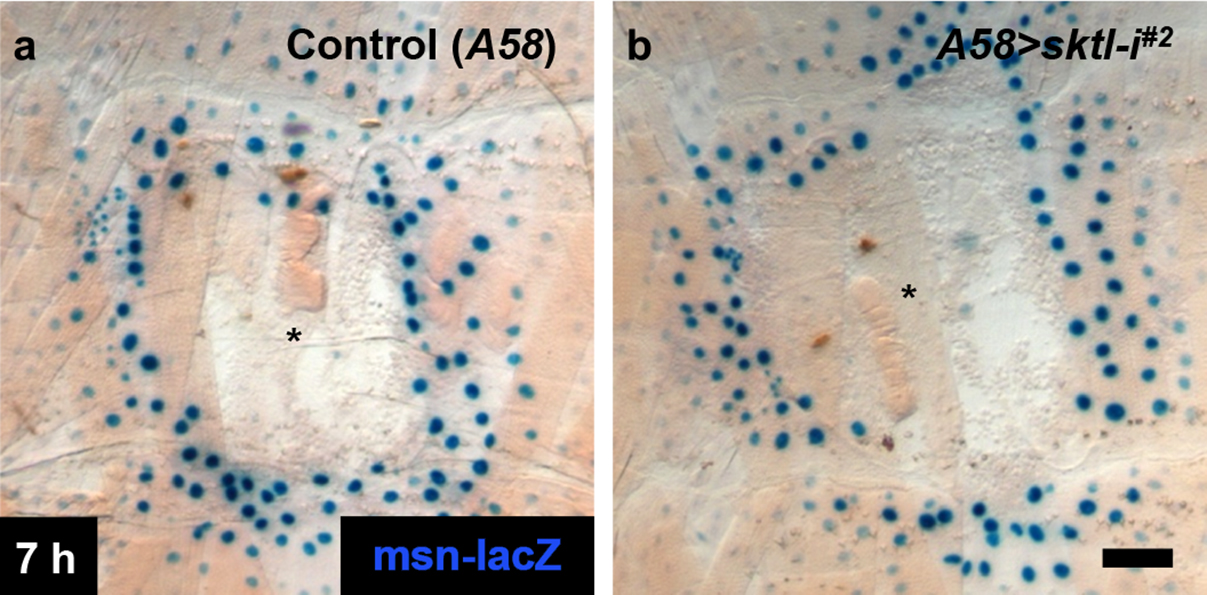
**

**Supplemental Figure S2. The JNK pathway was activated normally in wounded *sktl* knockdown larvae.** Activation of the JNK pathway was examined by the induction of *msn-lacZ* 7 h after wounding analysed by X-Gal staining in blue. (a) Controls. (b) *A58>sktl-i^#2^*. The asterisks indicate wound holes. Scale bar: 100 μm.

**
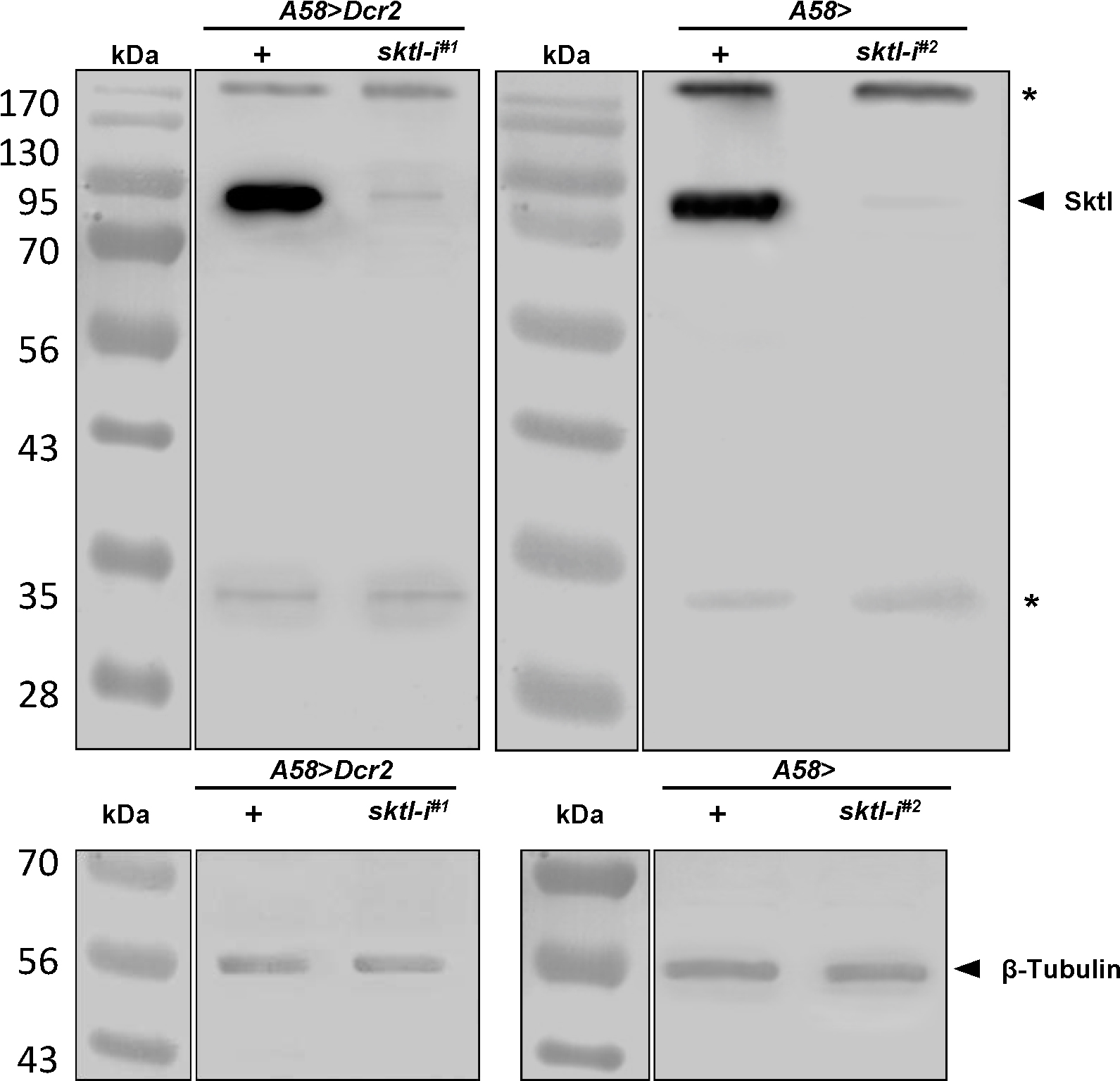
**

**Supplemental Figure S3 (Related to Figure 3j). Analysis of the knockdown efficiency of the two *sktl-RNAi* lines using Western blots.** Epidermal tissues of unwounded late 3rd instar larvae of the indicated genotypes were used. Anti-Sktl antibody was used to detect Sktl protein, and β-Tubulin was used as a loading control. The asterisks indicate non-specific bands.

**SUPPLEMENTAL METHODS**

**Analysis of β-galactosidase activity.** Larval epidermis was fixed in 2% glutaraldehyde for 15 min at room temperature. The sample was washed with PBS three times and incubated with 150 mM NaCl, 10 mM NaPO_4_, 3.1 mM K_4_[Fe_II_(CN)_6_], 3.1 mM K_3_[Fe_III_(CN)_6_], 1 mM MgCl_2_, 0.3% Triton X-100, and 0.2% X-Gal for 2 h at 37°C.

**SUPPLEMENTAL REFERENCES**

1 Pearn, M. T., Randall, L. L., Shortridge, R. D., Burg, M. G. & Pak, W. L. Molecular, biochemical, and electrophysiological characterization of Drosophila norpA mutants. *The Journal of biological chemistry* **271**, 4937-4945 (1996).
